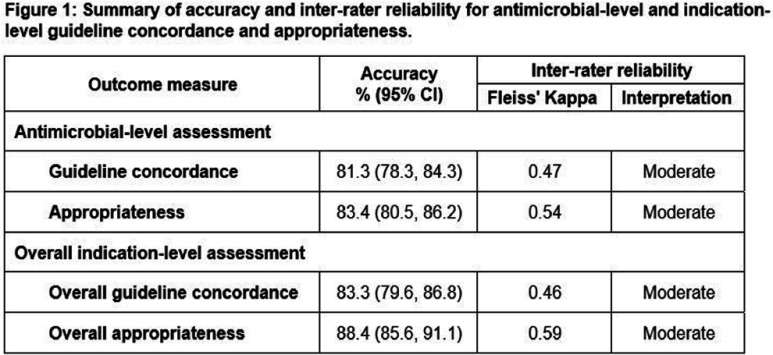# A validation study on a standardized assessment algorithm for antimicrobial prescribing appropriateness

**DOI:** 10.1017/ash.2025.266

**Published:** 2025-09-24

**Authors:** Caroline Chen, Josephine Wen, Courtney Ierano, Jenna Maleki, Tim Spelman, Rodney James, Karin Thursky, Lisa Hall

**Affiliations:** 1 National Centre for Antimicrobial Stewardship; The Royal Melbourne Hospital; 2 National Centre for Antimicrobial Stewardship; 3 National Centre for Antimicrobial Stewardship; 4 University of Melbourne; 5 The University of Queensland

## Abstract

**Background:** Since 2013, the Australian Hospital National Antimicrobial Prescribing Survey (Hospital NAPS) has provided a standardized framework for hospitals to assess the quality of antimicrobial prescribing. As part of the program’s continuous quality improvement, a revised appropriateness algorithm was developed and is scheduled for implementation in 2025. This study aims to validate this algorithm by evaluating accuracy and inter-rater reliability (IRR) in assessing guideline concordance and appropriateness. **Methods:** A prototype of the revised assessment algorithm was developed using Qualtrics®, including an assessment of antimicrobial-level guideline concordance, appropriateness and reasons for non-optimal prescribing, as well as overall indication-level guideline concordance and appropriateness. An eLearning module was developed to ensure consistency of training for assessors. Fourteen clinical vignettes (ten general and four specialist) across a range of real-world clinical scenarios and with varying levels of complexity were developed. Gold standard assessments were determined by an independent group of infectious diseases (ID) and antimicrobial stewardship (AMS) clinicians. Existing Hospital NAPS users were invited to participate. General vignettes were split into two equal groups and assigned to assessors in an alternating manner. Those with expertise in haematology/oncology or paediatrics were assigned additional specialist vignettes. Results were analyzed for accuracy against the gold standard, and for IRR using Fleiss’ Kappa coefficient. **Results:** A total of 102 assessors, across a range of professions, remoteness areas and years of auditing experience, completed their assigned vignettes. Assessors correctly identified the antimicrobial regimen for auditing in 91.9% of assessments, with incorrectly identified assessments excluded. A total of 681 antimicrobial-level and 534 indication-level assessments were analyzed. Figure 1 summarizes the accuracy and IRR for the main outcome measures of guideline concordance and appropriateness. Accuracy and IRR were higher for appropriateness compared with guideline concordance, and at the overall indication-level compared with the antimicrobial-level. Auditors correctly identified all gold-standard reasons for non-optimal prescribing in 68.3% of assessments. Across all measures, accuracy and IRR was higher amongst assessors with specialist ID/AMS experience compared to those without, from metropolitan compared with regional settings, and amongst those with 4 or more years of auditing experience. Pharmacists without ID/AMS expertise scored as highly as doctors and pharmacists with ID/AMS expertise. **Conclusion:** The revised Hospital NAPS algorithm provides a valid measure of guideline concordance and appropriateness. Higher accuracy and IRR were observed for appropriateness compared with guideline concordance, highlighting the importance of appropriateness as a measure for stewardship surveillance in reflecting quality of patient care.

**Figure:**